# Protein patterning by microcontact printing using pyramidal PDMS stamps

**DOI:** 10.1007/s10544-016-0036-4

**Published:** 2016-01-19

**Authors:** Luisa Filipponi, Peter Livingston, Ondřej Kašpar, Viola Tokárová, Dan V. Nicolau

**Affiliations:** Industrial Research Institute Swinburne, Faculty of Engineering and Industrial Science, Swinburne University of Technology, PO Box 218, VIC, 3122 Australia; Faculty of Engineering, Department of Bioengineering, McGill University, Macdonald Engineering Building, Room 378, 817 Sherbrooke Street West, Montreal, QC H3A 0C3 Canada

**Keywords:** Poly(dimethylsiloxane) microstructures, Protein patterning, Microcontact printing, Soft-lithography, Protein microarrays

## Abstract

Micro-contact printing, μCP, is a well-established soft-lithography technique for printing biomolecules. μCP uses stamps made of Poly(dimethylsiloxane), PDMS, made by replicating a microstructured silicon master fabricated by semiconductor manufacturing processes. One of the problems of the μCP is the difficult control of the printing process, which, because of the high compressibility of PDMS, is very sensitive to minute changes in the applied pressure. This over-sensitive response leads to frequent and/or uncontrollable collapse of the stamps with high aspect ratios, thus decreasing the printing accuracy and reproducibility. Here we present a straightforward methodology of designing and fabricating PDMS structures with an architecture which uses the collapse of the stamp to reduce, rather than enlarge the variability of the printing. The PDMS stamp, organized as an array of pyramidal micro-posts, whose ceiling collapses when pressed on a flat surface, replicates the structure of the silicon master fabricated by anisotropic wet etching. Upon application of pressure, depending on the size of, and the pitch between, the PDMS pyramids, an air gap is formed surrounding either the entire array, or individual posts. The printing technology, which also exhibits a remarkably low background noise for fluorescence detection, may find applications when the clear demarcation of the shapes of protein patterns and the distance between them are critical, such as microarrays and studies of cell patterning.

**Electronic supplementary material:**

The online version of this article (doi:10.1007/s10544-016-0036-4) contains supplementary material, which is available to authorized users.

## Introduction

Patterning proteins on surfaces have multiple applications in the area of biomedical microdevices, such as microarrays (Allison et al. 2006; Müller and Nicolau 2005), lab-on-a-chip (Chin et al. 2012), biosensors and bioMEMS (Mujahid et al. 2013), as well in the area of functional studies for cell and tissue development (Kane et al. 1999; Nicolau et al. 1996; Nicolau et al. 1999a). The patterning of proteins can be achieved by their immobilisation from solution in contact with surfaces presenting pre-fabricated patterns, using either selective covalent binding (Ivanova et al. 2002; Lenci et al. 2011; Nicolau et al. 1998, 1999b), or more rarely selective adsorption (Lan et al. 2005; Nicolau et al. 1999b). This strategy, which typically uses photolithographic techniques (Fodor et al. 1991), and even standard resist materials (Ivanova et al. 2002; Nicolau et al. 1998, 1999b), has the inherent advantage of high resolution of printing, at the expense of high cost of ownership and possible low signal/noise ratio due to the non-specific binding from protein solutions on areas outside the pattern of interest. Alternatively, protein patterns can be created by direct deposition methods, such as projection-based printing, e.g., ink-jet printing (Pardo et al. 2003), or based on laser microablation (Dobroiu et al. 2010), or contact-based printing, e.g., classical pin printing (Rowland et al. 2012), dip pen lithography (Huo et al. 2008), or microcontact printing, μCP (Bernard et al. 1998; Kumar et al. 1994). μCP has a special place in the panoply of direct deposition methods, because it is capable of very high resolution of printing, at a low cost of ownership, thus making it widely used in exploratory research (Delamarche et al. 2003; Delamarche et al. 1997; Kane et al. 1999; Renault et al. 2003). These advantages have led to attempts of applying μCP in many various applications, e.g., patterning neuronal cells (Nicolau et al. 1996; Thiebaud et al. 2002), fundamental studies of cell viability and growth as a function of available space (Amirpour et al. 2001; Chen et al. 1997; Ghosh et al. 2008; Nicolau et al. 1999a), and various microarray and lab-on-a-chip applications (Didar et al. 2012; Hoshino et al. 2014). Protein μCP can be applied to a large variety of substrates such as glass (plain or treated) (Bou Chakra et al. 2008), SAM-coated gold slides (Lee et al. 2008), and polymers, such as polystyrene (Bernard et al. 1998; Bernard et al. 2000).

Although there are several variations of μCP technology (Kaufmann and Ravoo 2010; Kim et al. 2008; Wolfe et al. 2010), all are based on a core process: (i) fabrication of a ‘master’, usually made of silicon using standard microlithography, which presents profiled topographies on its surface; (ii) casting a viscous pre-polymer, usually Poly(dimethylsiloxane), PDMS, on the master; (iii) hardening, by curing, of this “relief stamp” followed by the detachment from the master; (iv) immersion of the stamp in the solution of an ‘ink’, e.g., a solution of a protein; and finally (v) application of the stamp against a flat surface, thus selectively transferring the ink from the elevated features to the target surface (Bernard et al. 2000).

Despite these advantages, the central feature of the μCP, i.e., the high compressibility of PDMS, is also the cause of one of important disadvantages: the collapse of the stamp, which leads to deleterious features, e.g., irregular shapes and sizes of the patterns (Huang et al. 2005; Zhou et al. 2005). The present work proposes a design of the master comprising pyramidal wells, which offers a better management of the collapse of the stamp, and better lithographic parameters.

## Experimental details

### Fabrication of the silicon masters and PDMS stamps

#### Silicon master

The silicon master was fabricated by photolithography (spin coating the wafer with positive resist followed by pattern exposure and development), followed by anisotropic etching with a 28 % KOH solution along the <100 > crystallographic face of a silicon wafer, resulting in arrays of pyramidal wells (Ayeyard et al. 2010). The master comprised a variety of geometries, i.e., three series of arrays arranged in two rows, each including three arrays of 5 × 5 pyramidal wells. The wells forming the arrays in each row have same size (4 and 8 μm) but increasing inter-well distance (8, 16, 32 μm for 4 μm well size; and 10, 20, 40 μm for 8 μm well size; for detailed design of the silicon master see Supplementary Information – Fig. SI1). The following nomenclature will be used throughout the text; i.e., [number1 μm base]/[number2 μm pitch] representing [the pyramidal base size]/[pitch size between two pyramids]. The wafers were cut into 1 cm × 1 cm chips, each comprising 60 microarrays, and further used as masters for the replication of PDMS stamps.

#### PDMS stamp

The PDMS stamp was fabricated using polydimethylsiloxane (PDMS; Sylgard 184, Dow-Corning) following a well-known procedure (Duffy et al. 1998) with minor modifications. Briefly, ten parts of pre-polymer were mixed with one part of the cross-linker and degassed under vacuum for one hour. The mixture was then gently poured on top of the silicon master and cured for two hours at 65 °C. The cured polymer was then peeled off from the master and left at 85 °C overnight.

### Protein patterning

**Scheme 1:**
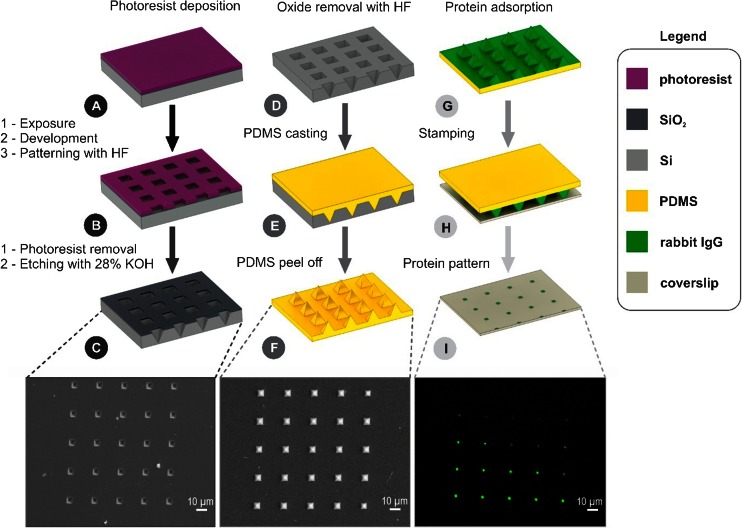
Graphical illustration and microscope images of silicon master fabrication (left column with SEM image), PDMS stamp preparation step (middle column with SEM image of the pyramidal features) and protein patterning steps (right column with fluorescent image of protein stamped on a glass slide)

Rabbit IgG (2 mg/ml in PBS 10 mM, Molecular Probes), was diluted to 50 μg/mL with PBS 10 mM (0.1 M phosphate buffer, 10 mM NaCl) and adsorbed over a freshly prepared PDMS stamp (100 μL) for 40 min. The stamp was subsequently rinsed thoroughly with PBS 10 mM (3 × 100 mL) and MilliQ water (3 × 100 mL), air dried and immediately stamped over a cleaned glass coverslip. Conformal contact was allowed to occur spontaneously and stamping was carried out for 60 s, at room temperature, without the use of additional pressure. Subsequently, the IgG-printed substrates have been incubated with a solution of secondary anti-rabbit IgG, Fluorescein labelled (same protocols as above) for 1 h. The fabrication of the Si masters, the PDMS stamps, and the μCP of protein patterns are presented in Scheme 1.

To compare the image quality of the μCP patterns and those obtained by classical microarrays, identical chemicals and solutions, (buffers, protein concentration, fluorophores) and processing conditions (humidity), have been also used to fabricate a microarray using an Inkjet robotic spotting MicroArraying System (PerkinElmer Piezorray) according to methodology described elsewhere (Abdo et al. 2005).

### Imaging and analysis of the masters, stamps and protein patterns

The AFM imaging and analysis was carried out on an Explorer system (Thermo Microscopes) in the normal contact mode, captured by the SPMLab Version 5.01 software. Images were obtained using an 8 μm Z, linearized scanner (100 μm × 100 μm) under air conditions of temperature of 23 °C and 45 % relative humidity. The AFM probes used for this study were pyramidal-tipped, silicon nitride cantilevers with a spring constant of 0.032 N/m. The image analysis was performed using the program WSxM V3.0 Beta 9.3 (Nanotec Electronica S.L.) with the topographical and lateral force images processed by subtraction of background and adjustment of brightness and contrast.

The collapsed stamps have been imaged with an inverted microscope (Olympus IX71), at 40× magnification. Protein fluorescent patterns have been imaged using the same microscope with epifluorescence optics (FITC filter setting), and mercury light source. Images were collected using a Coolview FDI high-resolution camera (Photonics Science Ltd.) controlled by Image-Pro Plus software (Ver. 5.0, Media Cybernetics). Image analysis of the fluorescent images allowed the calculations of the Signal/Noise (S/N).

## Results and discussion

### Analysis of the Si masters and PDMS stamps

The designs of the Si masters to be replicated in PDMS stamps comprise calibrated features in the expectation that the dimensions of the footprint of the stamps will be replicated in the final patterns. However, the collapse of the PDMS stamps results in deviations from the designed geometry either in size, or shape, or both. For contact printing, the aspect ratio of the PDMS features, described as the ratio of the height to the width, of the feature, respectively (Delamarche et al. 1997), is usually in the range of 0.2 to 2. For pyramidal wells, the aspect ratio can be defined as [height of the pyramid]/[width of the contact area during printing]. By applying different printing pressure, the width of the contact area increases and the height of the pyramid decreases (Hong et al. 2008). The design of Si masters presenting pyramidal wells has been used before (Filipponi et al. 2009; Hong et al. 2008; Schwinger et al. 2008), largely as a method to achieve very high resolution of the patterns without the high cost of ownership, or the need to fabricate very fine features in the Si master for PDMS replication.

**Fig. 1:**
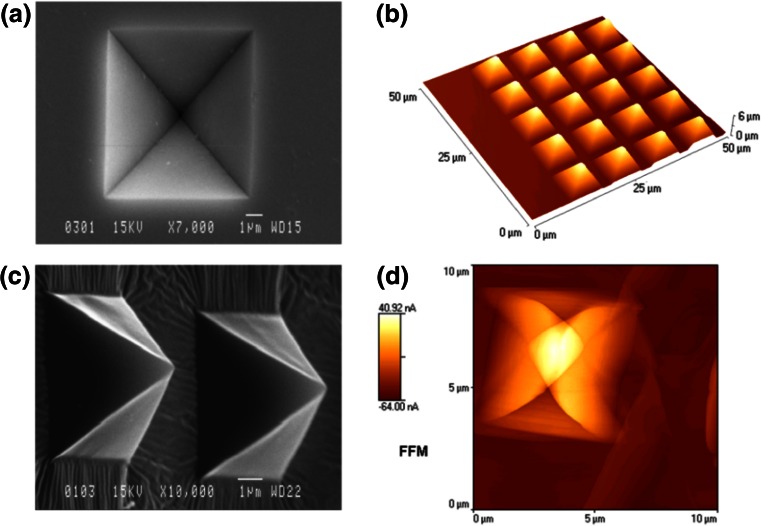
Imaging and analysis of the silicon masters and PDMS stamps. Left panel: SEM images of a well in the Si master (**a**) and of a PDMS pyramid (**c**). Right panel: AFM imaging and analysis of PDMS stamps. AFM imaging of an array of PDMS pyramids (**b**). Lateral Force Mode (LFM) scanning of an individual PDMS pyramid. While the topography of the PDMS structures presents a nearly perfect pyramidal shape (**b**), the analysis by LFM reveals the existence of a soft tip at the apex of the pyramid, which reversibly bends when in contact with the AFM tip (**d**)

**Fig. 2:**
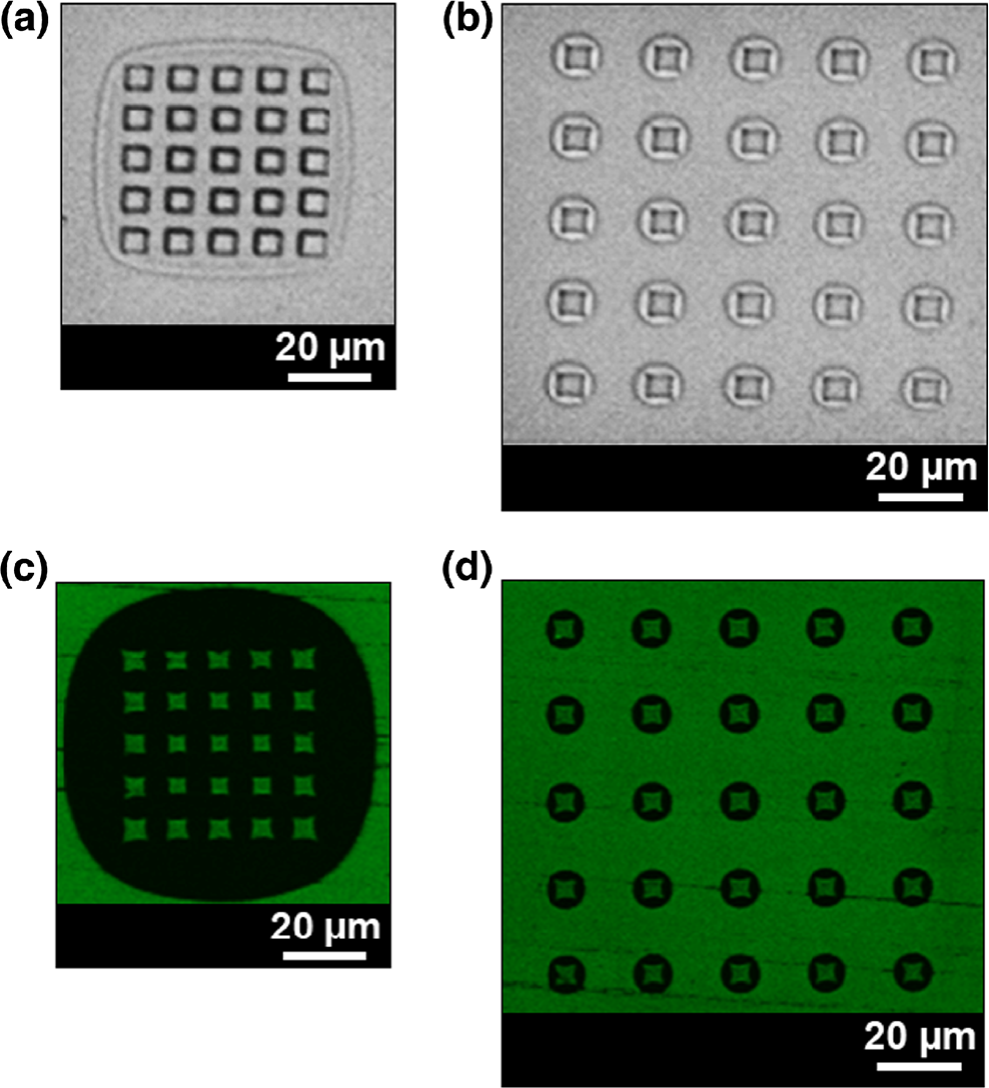
*Top*: Transmission images of controlled collapse of all size classes of the PDMS stamps for pyramidal features [4 μm base/8 μm pitch] (**a**) and [4 μm base/16 μm pitch] (**b**). The inter-well distance together with pyramidal dimensions result in air being trapped around the whole arrays (**a** and **c**), or around individual pyramids (**b** and **d**). *Bottom*: Epi-fluorescent images of two protein arrays, with the same dimensions as (**a**) and (**b**), fabricated via μCP

The pyramidal wells replicate in the PDMS stamps with excellent fidelity, i.e., with less than 5 % variation between the dimensions of the master and those of the replica. Size analysis of the PDMS pyramids was confirmed by the AFM measurement as in agreement with the designed dimensions of the master (summarised in Tab. SI 1 of the Supporting Information). The aspect ratio of the PDMS stamps, as defined above, ranges from 22.5 when no pressure is applied on the stamp (Fig. 1) to 1.3 after collapse of pyramids in the process of printing (Fig. 2).

AFM analysis, performed by Lateral Force Mode (LFM), offers more insight into the nano-mechanics of the material of the PDMS stamp (Fig. 1d). LFM has been used to identify and localise different states and/or compositions of materials patches at nm-level, e.g., hybridization of DNA (Nicolau et al. 2005), localisation of protein immobilisation on polymers (Nicolau et al. 2010), composition of hair (Smith and Swift 2002), and, importantly, the morphology of polymer blends (Tsou and Waddell 2002; Žukiene et al. 2006). Recently it has been shown (Nikogeorgos et al. 2012) that the AFM contact mechanics are best modelled by treating the friction force as the sum of a load-dependent term (attributed to “ploughing”) and an area-dependent term (attributed to shearing, i.e., adsorption). Because the PDMS stamps are chemically-homogenous, and because the slope of their surface is constant, the LFM will only ‘read’ the local mechanical properties of the stamp, via a “ploughing” factor (Nikogeorgos et al. 2012).

### PDMS stamp collapse

For elastomeric stamps, such as PDMS, which presents a high compressibility (Young’s modulus ≈ 3 MPa (Bietsch and Michel 2000)); and for features with high aspect ratios, the collapse of the stamp is an inherent process. This process often leads to deleterious effects, e.g., deviations in size and shape from the intended pattern, variations of concentrations in various parts of the printed area, and even printing in areas that should have be ink-free. Finally, the collapse of the stamp can take various forms (Sharp et al. 2004): (i) roof collapse of low aspect ratio recesses (Decré et al. 2005), (ii) buckling of high aspect ratio plates (Hui et al. 2002), and (iii) lateral sticking of high aspect ratio plates (Bietsch and Michel 2000), each with their specific negative impacts on the quality of printing. Because of its importance, the collapse of the PDMS stamps has been thoroughly investigated (Bietsch and Michel 2000; Decré et al. 2005; Hui et al. 2002; Jagota et al. 2002; Petrzelka and Hardt 2012). As described elsewhere (Delamarche et al. 1997), in order to avoid or mitigate the unwanted structural deformations during μCP, the aspect ratio of PDMS features has to be in the range of 0.2 to 2, and the inter-feature distance should not exceed 20 times their height. While the pyramidal profile of the PDMS stamps have been used occasionally in the past (Filipponi et al. 2009; Hong et al. 2008; Schwinger et al. 2008), most of the above mentioned analyses refer to rectangular, or cylindrical profiles of the stamps.

Similarly to previous studies using rectangular (or cylindrical) PDMS pillar, when collapsing occurs, the base plane of the PDMS pattern makes contact with the substrate, and an air gap is trapped between the base plane and the printing substrate (Bietsch and Michel 2000). Depending on the size of the PDMS and the distance between them, the collapse of the stamp results in two types of architectures. If the distance between the pyramids is large enough, e.g., 16 μm and 40 μm for pyramidal structures with 4 μm and 8 μm base size, respectively, the roof collapses around the rectangular imprint of the pyramidal tip. Conversely, when the distance between pyramids is small enough, e.g., 8 μm for pyramidal base of 4 μm and 10 and 20 μm for 8 μm base size, the roof collapses around the group of PDMS features (Fig. SI 2).

The use of the pyramidal architecture for the PDMS stamps has several advantages. First, the fabrication of the silicon masters is straightforward, as wet anisotropic etching avoids the use of Reactive Ion Etching, and it can be performed in essentially any laboratory. Second, and more importantly, the pyramidal shape of the stamp makes the collapse by buckling and by lateral sticking, i.e., two of the main collapse modes of PDMS stamps (Sharp et al. 2004), irrelevant. Third, the confinement of the softer material at the apex of the pyramids, as indicated by LFM analysis, has the potential to self-regulate the pattern size. Finally, the air trapped either around individual, or around groups of PDMS pillars, clearly demarcates the stamped patterns, thus potentially making their quantification easier.

### μCP

PDMS is a highly hydrophobic elastomer, which efficiently adsorbs proteins from solution. Once the stamp is “inked” with a protein solution and placed in conformal contact with a surface, the protein layer is transferred from the stamp to the receiving substrate. During μCP, an air gap is formed around the densely packed arrays, and the result is an array of protein patterns with very sharp contours on a background with essentially no fluorescence. Consequently, the signal/noise ratio is very high. Figure 2 presents μCP protein patterns over a glass substrate using a protein solution of fluorescein-labelled anti-rabbit IgG.

These results suggest how μCP can be further used to pattern protein- or, more generally- biomolecule microarrays, where the single elements of the array are either individually separated, or enclosed in an area having same surface properties. The incubation of the patterned array with a second biomolecule solution would allow the fabrication of multiple patterns that could be useful in studies where biomolecule co-patterns are required, e.g., cell patterning.

### Comparison between μCP and ink jet-printed spots

Robotic spotting is one of the methods currently employed for the fabrication of protein microarrays, since it allows fast printing over large surfaces using pL quantities of protein solutions. Unfortunately, the method often leads to uneven adsorption of the protein within the spotted area, which is a results of a combination of effects, i.e., the temperature of the substrate, solution and printing environment, the viscosity of the solution, the type of protein spotted, and the type of substrate used, just to mention few. This creates spots having a typical ‘donut shape’. Also, surfaces that allow uniform and global attachment of a wide variety of proteins are not currently available, therefore the method requires optimizing the type of substrate used for the specific protein to be printed.

μCP has been used to print a wide range of proteins over large areas with bioactivity retention and is therefore a possible alternative to robotic spotting for the fabrication of protein microarrays. In this context, μCP with pyramidal PDMS features allows the fabrication of arrays where its individual elements are similar in shape and intensity.

**Fig. 3:**
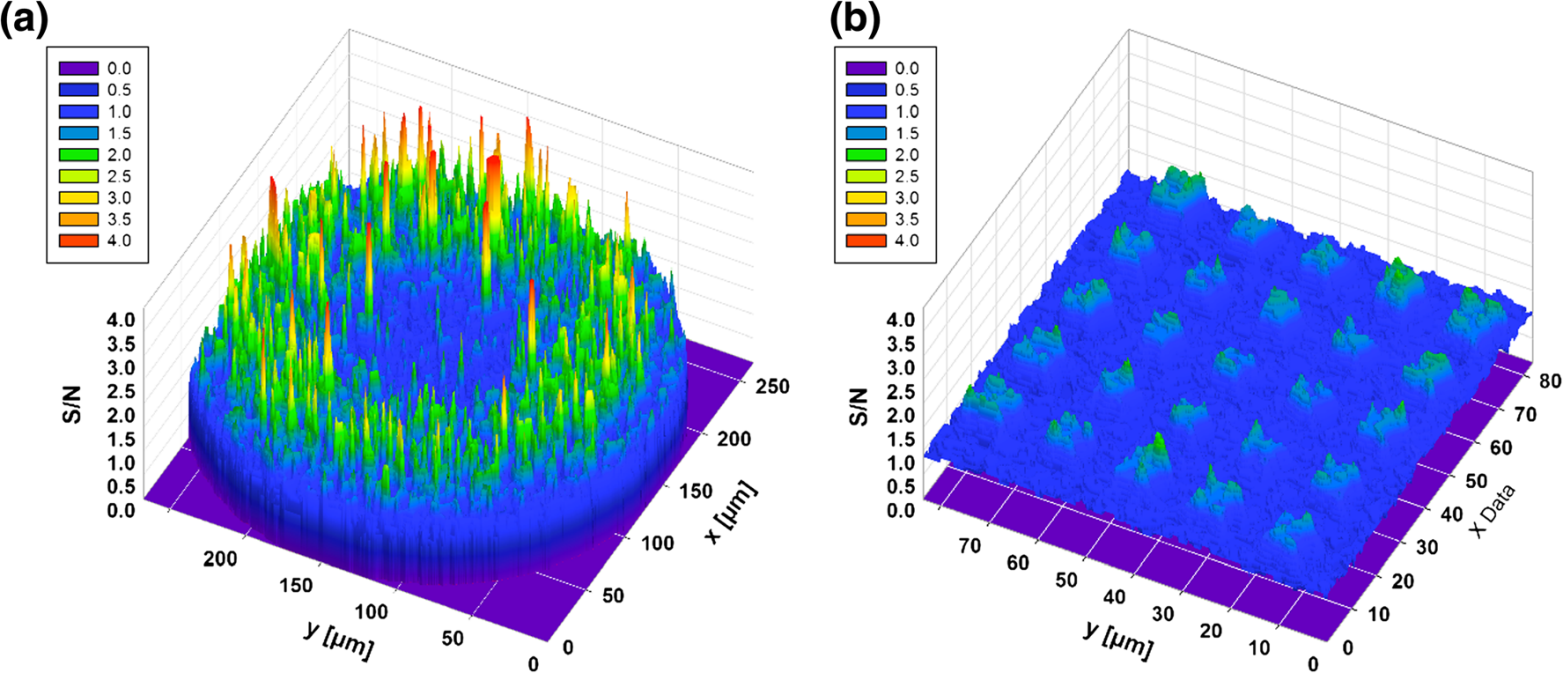
S/N 3D surface plot of a robotically printed spot (250 μm wide) (**a**) and a μCP array [8 μm base/20 μm pitch] (**b**)

While the mean values of S/N are comparable (i.e., 1.42 for inkjet printing, and 1.54 for μCP), the standard deviation, and the variance of the fluorescence signal on a classical, ink jet-printed spot is considerably higher, i.e., 3× and 9×, respectively, than that of the array printed by μCP, for identical operational conditions, e.g., proteins, concentrations, washing, humidity, etc. (Fig. SI 3). This is also confirmed by 3D S/N graph visualization (Fig. 3).

## Conclusions

A PDMS stamp having pyramid-like relief microfeatures organized in arrays collapses when placed over a flat substrate, with the consequent formation of a large air gap around the entire array or around each post composing the array, depending on the distance between the posts. A method that exploits the collapsing of such a PDMS pattern is presented. When high-density arrays are used, μCP allows the printing of patterns of labelled proteins in an array format, where the elements of the array have similar shape and similar fluorescent signal, with excellent signal-to-noise ratio. The method could be used for the fabrication of protein microarrays that are often fabricated with a robotic spotting method, frequently leading to spots having uneven protein adsorption.

## Electronic Supplementary Material

ESM 1 (DOCX 1.64 mb)
