# Supplementary material for: Protein patterning by microcontact printing using pyramidal PDMS stamps
Source: Biomed Microdevices. 2016 Jan 19;18:9. doi: 10.1007/s10544-016-0036-4 (PMC4718951; doi:10.1007/s10544-016-0036-4)
Supplement: Supplementary file 1 — (DOCX 1.64 mb) [file 10544_2016_36_MOESM1_ESM.docx]

**Supplementary Information**

**Protein patterning by microcontact printing using pyramidal PDMS stamps**

*Luisa Filipponi^1^, Peter Livingston^1^, Ondřej Kašpar^2^, Viola Tokárová^2^, Dan V. Nicolau^1, 2^**

^1^ Industrial Research Institute Swinburne, Faculty of Engineering and Industrial Science, Swinburne University of Technology, PO Box 218, VIC 3122, Australia

^2^ McGill University, Faculty of Engineering, Department of Bioengineering, Montreal, Quebec, H3A 0C3, Canada

**SI 1. Design of the Si master**

The silicon master used for PDMS molding comprises three series of arrays arranged in two rows, R1 and R2. Each row includes five arrays of etched V-shaped holes arranged in a 5x5 matrix, which have holes of same size, but with increasing inter-well distance. The dimensions of the etched holes are 4 and 8 µm for row R1 and R2, respectively. The inter-well distance increases along the row (8/16/32 µm for R1 and 10/20/40 µm for R2).


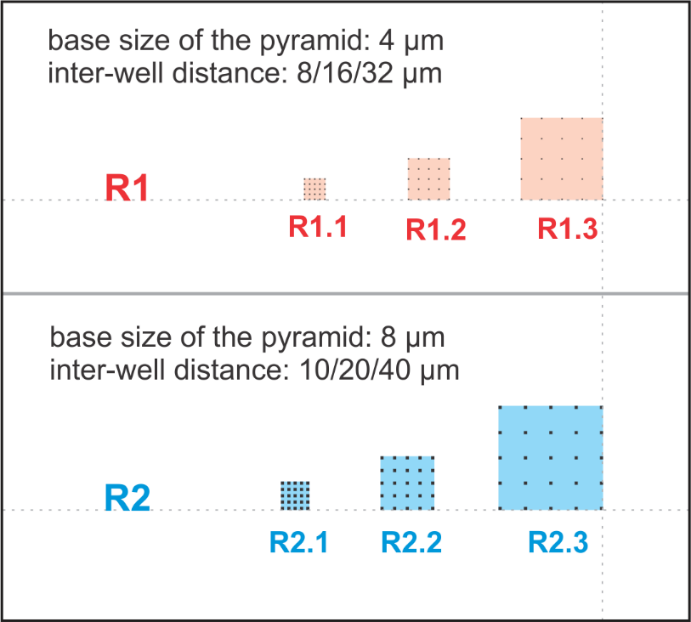


**Fig. SI 1** Design of the silicon master for µCP using pyramidal stamps

The measured width of pyramidal structure is 3.6, and 7.8 µm; and the height is 2.7 and 4.9 µm for pyramids R1 and R2, respectively.

**Tab. SI 1** Size analysis of the pyramids – design vs. AFM measurements

| Structure type | Width -design (µm) | Width –AFM (µm) | Height – calculated (µm) | Height –AFM (µm) | Inter-well distance (µm) |
| --- | --- | --- | --- | --- | --- |
| R1.1 | 4.0 | 3.6 | 3.0 | 2.7 | 8.0 |
| R1.2 | 4.0 | 3.6 | 3.0 | 2.7 | 16.0 |
| R1.3 | 4.0 | 3.6 | 3.0 | 2.7 | 32.0 |
| R2.1 | 8.0 | 7.8 | 5.5 | 4.9 | 10.0 |
| R2.2 | 8.0 | 7.8 | 5.5 | 4.9 | 20.0 |
| R2.3 | 8.0 | 7.8 | 5.5 | 4.9 | 40.0 |

**
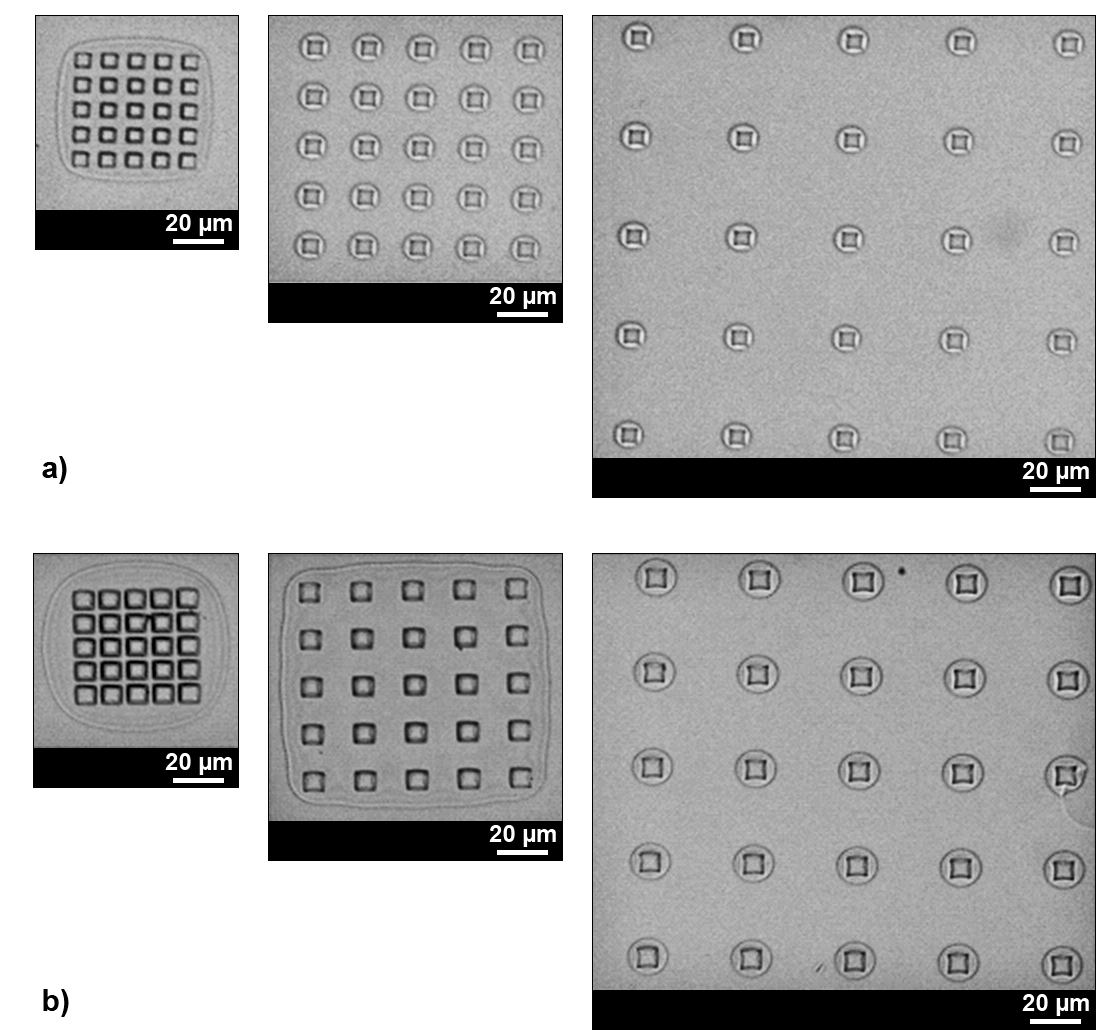
**

**Fig. SI 2** The interplay between the size of the pyramids and the inter-well distance results in the air being trapped either around an array, e.g., geometries [4 µm base/8 µm pitch], [8 µm base/10 µm pitch] and [8 µm base/20 µm pitch]; or around individual pyramids, e.g., [4 µm base/16 µm pitch], [4 µm base/32 µm pitch] and [8 µm base/40 µm pitch]

**
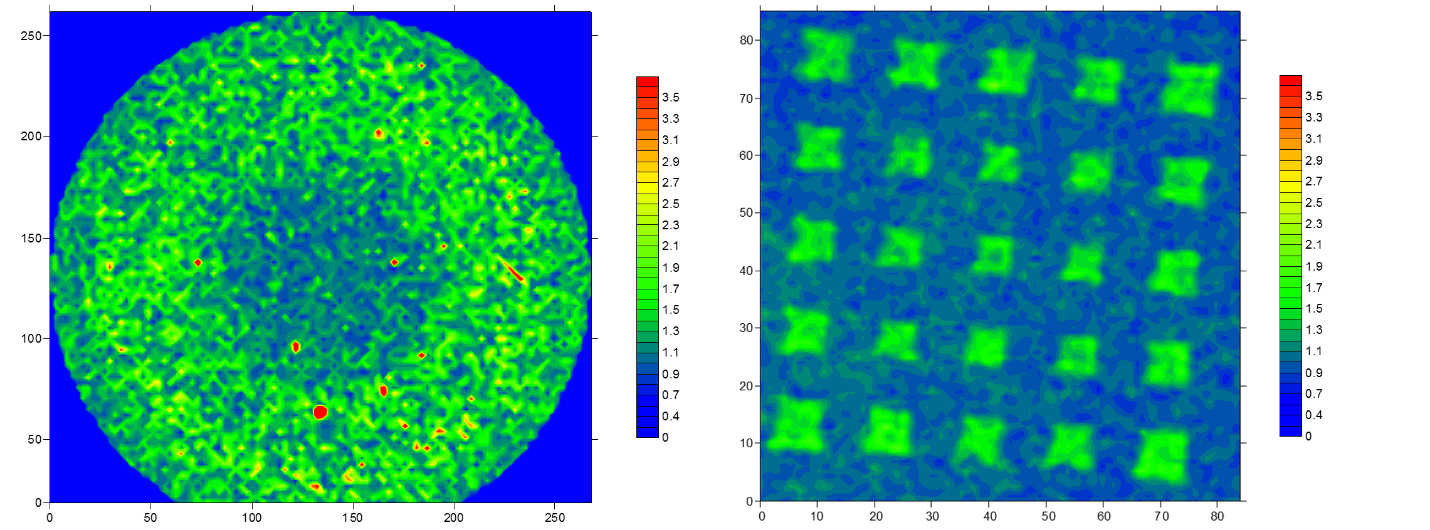
**

**Fig. SI 3** S/N Contour plot of a robotically printed spot (250 µm wide) (left) and a µCP array [8 µm base/20 µm pitch] (right)
